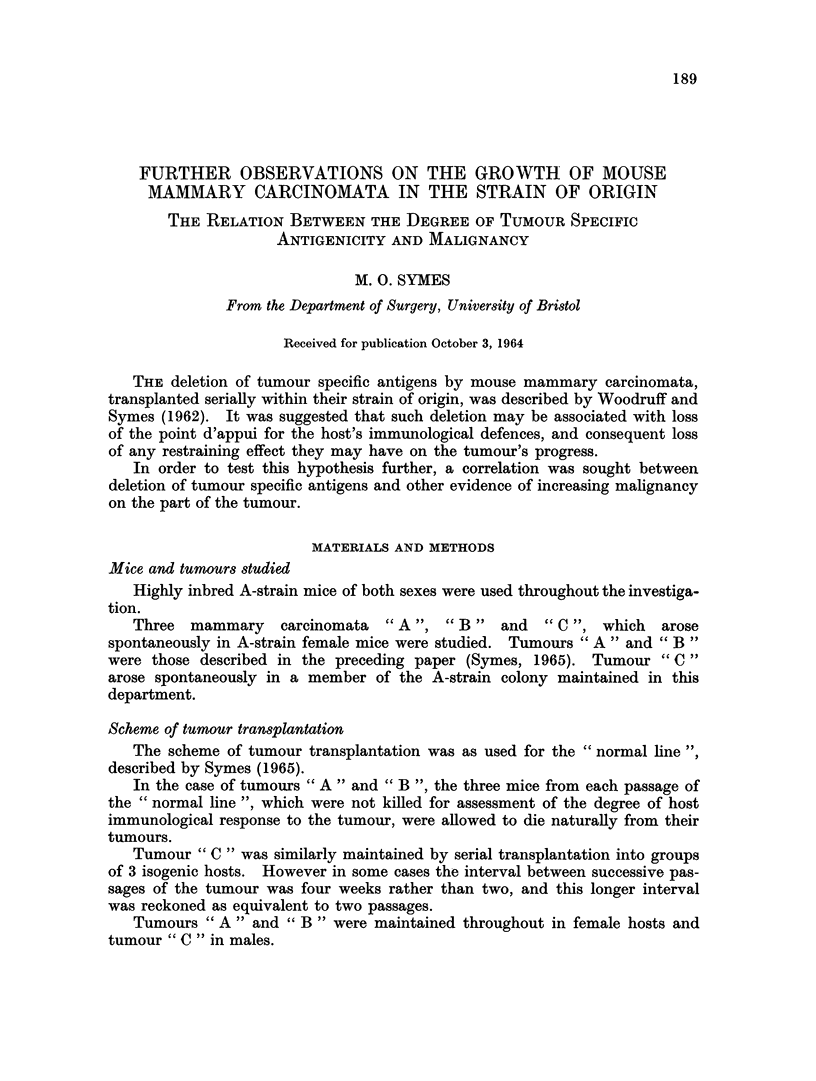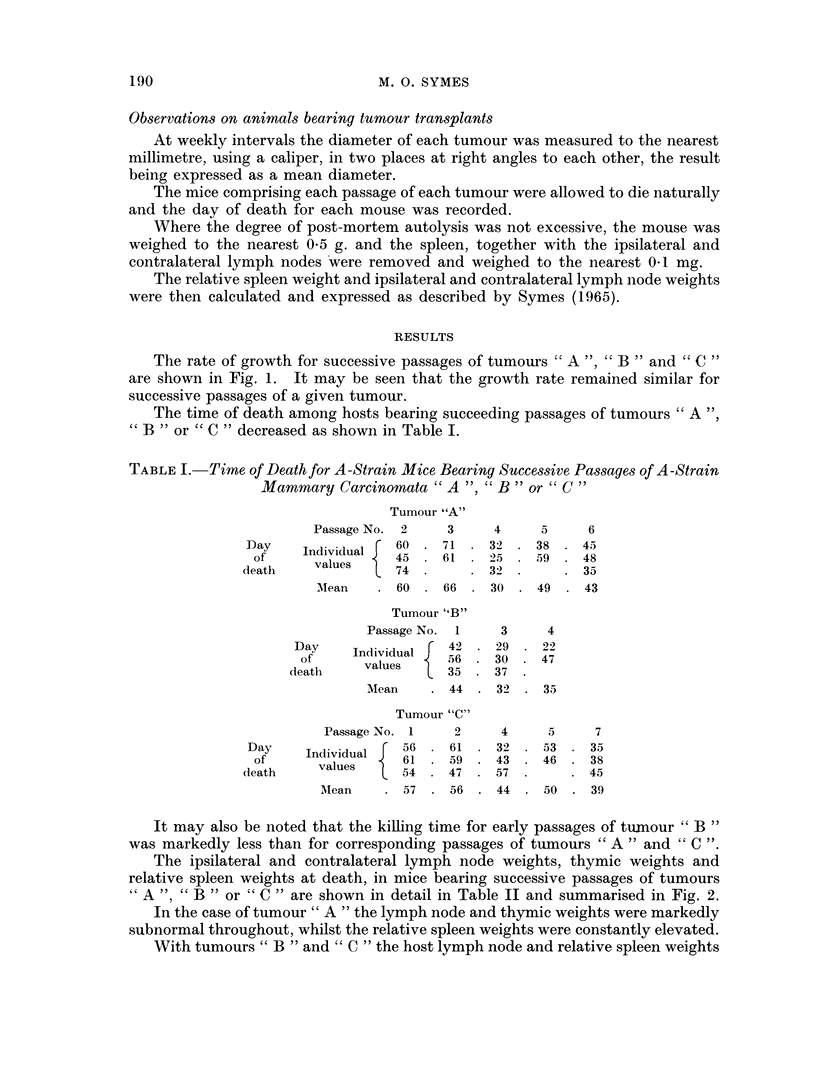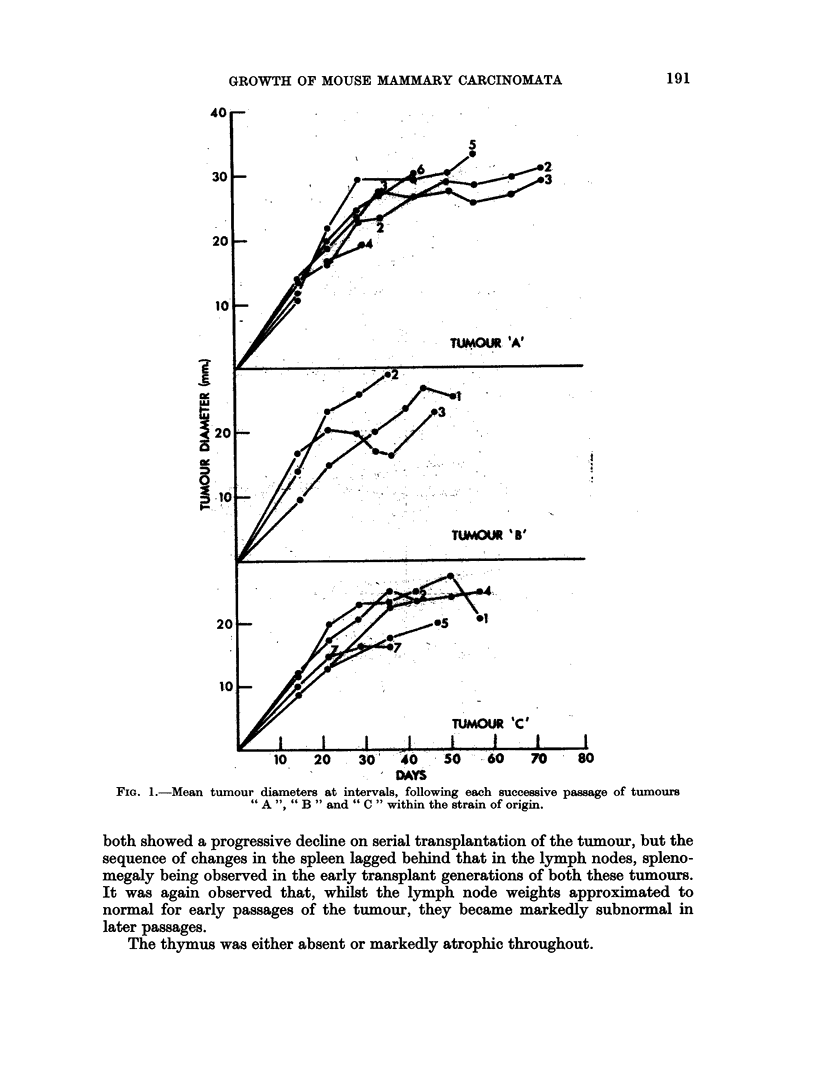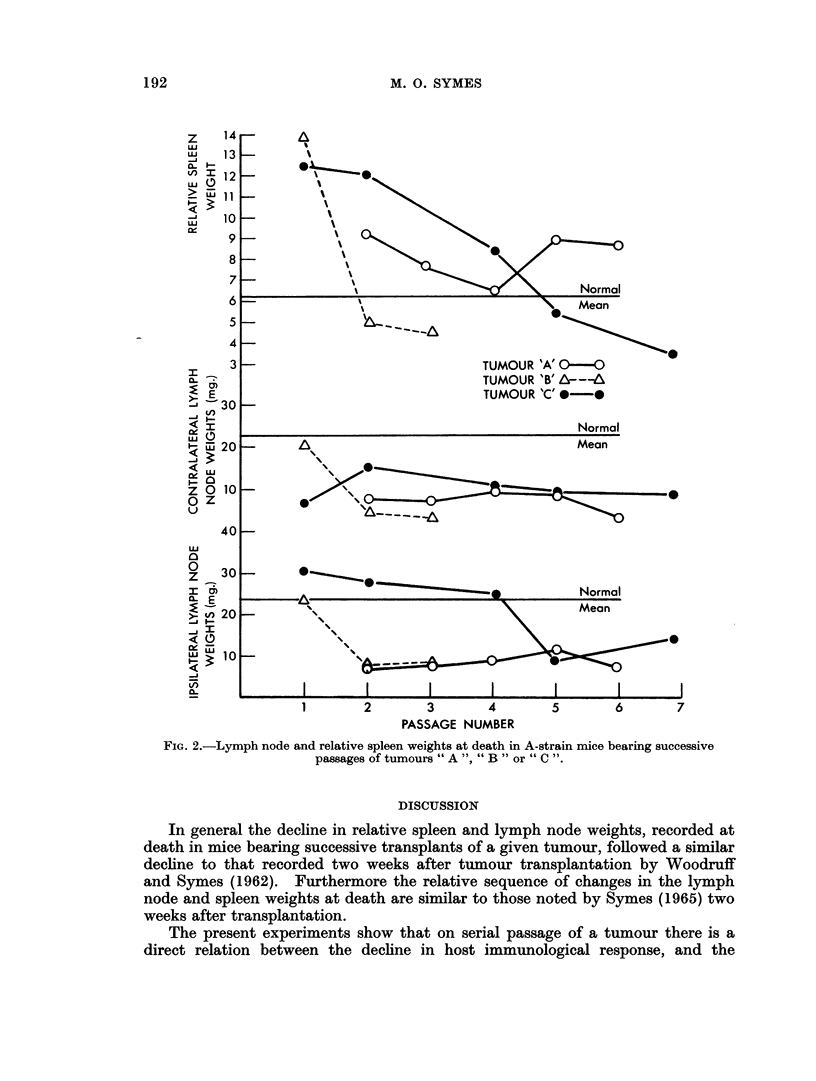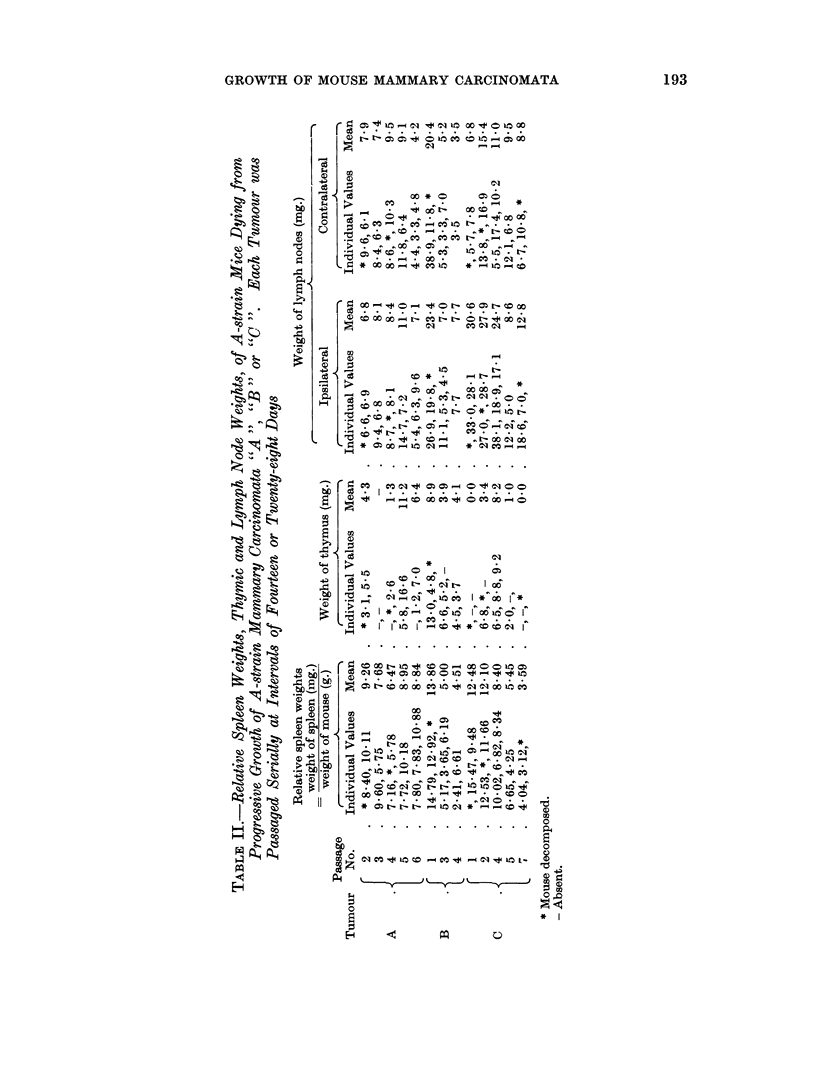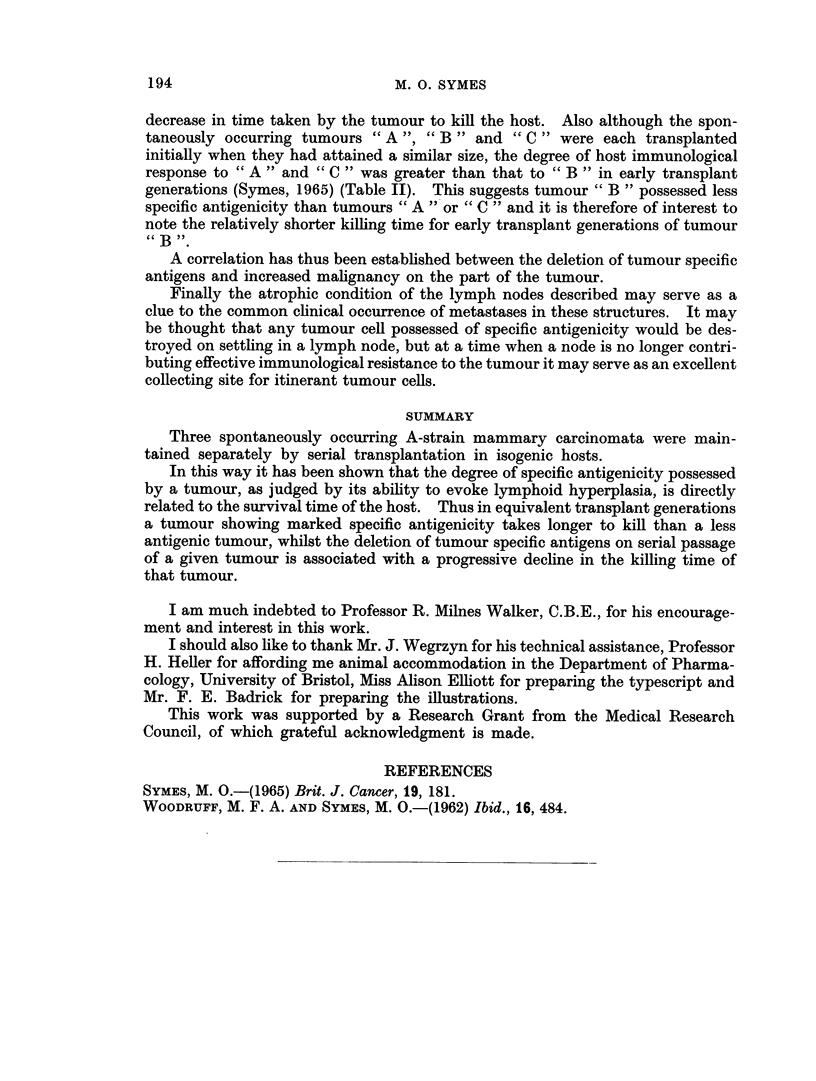# Further Observations on the Growth of Mouse Mammary Carcinomata in the Strain of Origin

**Published:** 1965-03

**Authors:** M. O. Symes


### FURTHER OBSERVATIONS ON THE GROWTH OF MOUSE MAMMARY CARCINOMATA IN THE STRAIN OF ORIGIN

### The Relation Between the Degree of Tumour Specific Antigenicity and Malignancy

M. O. SYMES

*From the Department of Surgery, University of Bristol*

Received for publication October 3, 1964

THE deletion of tumour specific antigens by mouse mammary carcinomata, transplanted serially within their strain of origin, was described by Woodruff and Symes (1962). It was suggested that such deletion may be associated with loss of the point d'appui for the host's immunological defences, and consequent loss of any restraining effect they may have on the tumour's progress.

In order to test this hypothesis further, a correlation was sought between deletion of tumour specific antigens and other evidence of increasing malignancy on the part of the tumour.

### MATERIALS AND METHODS

*Mice and tumours studied*

Highly inbred A-strain mice of both sexes were used throughout the investigation.

Three mammary carcinomata " A ", " B " and " C ", which arose spontaneously in A-strain female mice were studied. Tumours " A " and " B " were those described in the preceding paper (Symes, 1965). Tumour " C " arose spontaneously in a member of the A-strain colony maintained in this department.

*Scheme of tumour transplantation*

The scheme of tumour transplantation was as used for the " normal line ", described by Symes (1965).

In the case of tumours " A " and " B ", the three mice from each passage of the " normal line ", which were not killed for assessment of the degree of host immunological response to the tumour, were allowed to die naturally from their tumours.

Tumour " C " was similarly maintained by serial transplantation into groups of 3 isogenic hosts. However in some cases the interval between successive passages of the tumour was four weeks rather than two, and this longer interval was reckoned as equivalent to two passages.

Tumours " A " and " B " were maintained throughout in female hosts and tumour " C " in males.

*Observations on animals bearing tumour transplants*

At weekly intervals the diameter of each tumour was measured to the nearest millimetre, using a caliper, in two places at right angles to each other, the result being expressed as a mean diameter.

The mice comprising each passage of each tumour were allowed to die naturally and the day of death for each mouse was recorded.

Where the degree of post-mortem autolysis was not excessive, the mouse was weighed to the nearest 0·5 g. and the spleen, together with the ipsilateral and contralateral lymph nodes were removed and weighed to the nearest 0·1 mg.

The relative spleen weight and ipsilateral and contralateral lymph node weights were then calculated and expressed as described by Symes (1965).

### RESULTS

The rate of growth for successive passages of tumours " A ", " B " and " C " are shown in Fig. 1. It may be seen that the growth rate remained similar for successive passages of a given tumour.

The time of death among hosts bearing succeeding passages of tumours " A ", " B " or " C " decreased as shown in Table I.

TABLE I.—*Time of Death for A-Strain Mice Bearing Successive Passages of A-Strain Mammary Carcinomata " A ", " B " or " C "*

Tumour "A"

| | Passage No. | 2 | 3 | 4 | 5 | 6 |
|---|---|---|---|---|---|---|
| Day of death | Individual values | 60 | 71 | 32 | 38 | 45 |
| | | 45 | 61 | 25 | 59 | 48 |
| | | 74 | | 32 | | 35 |
| | Mean | 60 | 66 | 30 | 49 | 43 |

Tumour "B"

| | Passage No. | 1 | 3 | 4 |
|---|---|---|---|---|
| Day of death | Individual values | 42 | 29 | 22 |
| | | 56 | 30 | 47 |
| | | 35 | 37 | |
| | Mean | 44 | 32 | 35 |

Tumour "C"

| | Passage No. | 1 | 2 | 4 | 5 | 7 |
|---|---|---|---|---|---|---|
| Day of death | Individual values | 56 | 61 | 32 | 53 | 35 |
| | | 61 | 59 | 43 | 46 | 38 |
| | | 54 | 47 | 57 | | 45 |
| | Mean | 57 | 56 | 44 | 50 | 39 |

It may also be noted that the killing time for early passages of tumour " B " was markedly less than for corresponding passages of tumours " A " and " C ".

The ipsilateral and contralateral lymph node weights, thymic weights and relative spleen weights at death, in mice bearing successive passages of tumours " A ", " B " or " C " are shown in detail in Table II and summarised in Fig. 2.

In the case of tumour " A " the lymph node and thymic weights were markedly subnormal throughout, whilst the relative spleen weights were constantly elevated.

With tumours " B " and " C " the host lymph node and relative spleen weights

FIG. 1.—Mean tumour diameters at intervals, following each successive passage of tumours " A ", " B " and " C " within the strain of origin.

both showed a progressive decline on serial transplantation of the tumour, but the sequence of changes in the spleen lagged behind that in the lymph nodes, splenomegaly being observed in the early transplant generations of both these tumours. It was again observed that, whilst the lymph node weights approximated to normal for early passages of the tumour, they became markedly subnormal in later passages.

The thymus was either absent or markedly atrophic throughout.

FIG. 2.—Lymph node and relative spleen weights at death in A-strain mice bearing successive passages of tumours " A ", " B " or " C ".

### DISCUSSION

In general the decline in relative spleen and lymph node weights, recorded at death in mice bearing successive transplants of a given tumour, followed a similar decline to that recorded two weeks after tumour transplantation by Woodruff and Symes (1962). Furthermore the relative sequence of changes in the lymph node and spleen weights at death are similar to those noted by Symes (1965) two weeks after transplantation.

The present experiments show that on serial passage of a tumour there is a direct relation between the decline in host immunological response, and the decrease in time taken by the tumour to kill the host. Also although the spontaneously occurring tumours " A ", " B " and " C " were each transplanted initially when they had attained a similar size, the degree of host immunological response to " A " and " C " was greater than that to " B " in early transplant generations (Symes, 1965) (Table II). This suggests tumour " B " possessed less specific antigenicity than tumours " A " or " C " and it is therefore of interest to note the relatively shorter killing time for early transplant generations of tumour " B ".

A correlation has thus been established between the deletion of tumour specific antigens and increased malignancy on the part of the tumour.

Finally the atrophic condition of the lymph nodes described may serve as a clue to the common clinical occurrence of metastases in these structures. It may be thought that any tumour cell possessed of specific antigenicity would be destroyed on settling in a lymph node, but at a time when a node is no longer contributing effective immunological resistance to the tumour it may serve as an excellent collecting site for itinerant tumour cells.

### SUMMARY

Three spontaneously occurring A-strain mammary carcinomata were maintained separately by serial transplantation in isogenic hosts.

In this way it has been shown that the degree of specific antigenicity possessed by a tumour, as judged by its ability to evoke lymphoid hyperplasia, is directly related to the survival time of the host. Thus in equivalent transplant generations a tumour showing marked specific antigenicity takes longer to kill than a less antigenic tumour, whilst the deletion of tumour specific antigens on serial passage of a given tumour is associated with a progressive decline in the killing time of that tumour.

I am much indebted to Professor R. Milnes Walker, C.B.E., for his encouragement and interest in this work.

I should also like to thank Mr. J. Wegrzyn for his technical assistance, Professor H. Heller for affording me animal accommodation in the Department of Pharmacology, University of Bristol, Miss Alison Elliott for preparing the typescript and Mr. F. E. Badrick for preparing the illustrations.

This work was supported by a Research Grant from the Medical Research Council, of which grateful acknowledgment is made.

### REFERENCES

Symes, M. O.—(1965) *Brit. J. Cancer*, **19**, 181.

Woodruff, M. F. A. and Symes, M. O.—(1962) *Ibid.*, **16**, 484.